# Recent Advances on Feasible Strategies for Monoterpenoid Production in *Saccharomyces cerevisiae*

**DOI:** 10.3389/fbioe.2020.609800

**Published:** 2020-12-01

**Authors:** Qiyu Gao, Luan Wang, Maosen Zhang, Yongjun Wei, Wei Lin

**Affiliations:** ^1^Department of Microbiology and Immunology, School of Medicine & Holistic Integrative Medicine, Nanjing University of Chinese Medicine, Nanjing, China; ^2^State Key Laboratory of Natural Medicines, China Pharmaceutical University, Nanjing, China; ^3^Jiangsu Collaborative Innovation Center of Chinese Medicinal Resources Industrialization, Nanjing, China; ^4^Key Laboratory of Advanced Drug Preparation Technologies, Ministry of Education and School of Pharmaceutical Sciences, Zhengzhou University, Zhengzhou, China; ^5^The Affiliated Hospital of Nanjing University of Chinese Medicine, Jiangsu Province Hospital of Chinese Medicine, Nanjing, China

**Keywords:** monoterpenoids, biosynthetic pathway, *Saccharomyces cerevisiae*, protein engineering, structure biology, synthetic biology

## Abstract

Terpenoids are a large diverse group of natural products which play important roles in plant metabolic activities. Monoterpenoids are the main components of plant essential oils and the active components of some traditional Chinese medicinal herbs. Some monoterpenoids are widely used in medicine, cosmetics and other industries, and they are mainly obtained by plant biomass extraction methods. These plant extraction methods have some problems, such as low efficiency, unstable quality, and high cost. Moreover, the monoterpenoid production from plant cannot satisfy the growing monoterpenoids demand. The development of metabolic engineering, protein engineering and synthetic biology provides an opportunity to produce large amounts of monoterpenoids eco-friendly using microbial cell factories. This mini-review covers current monoterpenoids production using *Saccharomyces cerevisiae*. The monoterpenoids biosynthetic pathways, engineering of key monoterpenoids biosynthetic enzymes, and current monoterpenoids production using *S. cerevisiae* were summarized. In the future, metabolically engineered *S. cerevisiae* may provide one possible green and sustainable strategy for monoterpenoids supply.

## Introduction

Terpenoids are the largest and most structurally diverse class of natural products widely distributed in plants, microorganisms, and insects. More than 55,000 terpenoids have been identified so far (Christianson, 2008). Terpenoids have diverse biological activities, and they can function as chemical defense agents against predation and anti-pathogenic agents (Hijaz et al., 2016; Mahizan et al., 2019). Many terpenoids play critical roles in the interactions of plant-plant and plant-environment (Abbas et al., 2017). Among them, monoterpenoids are a kind of terpenoids composed with two isoprene units, which are widely distributed in plant and used in both pharmacy and medicine (Wojtunik-Kulesza et al., 2019). Recently, sabinene, one kind of monoterpene, has the great potential to be used as advanced biofuel (Zhang et al., 2014). Nowadays, monoterpenoids are mainly extracted from plants using chemical methods. The monoterpenoid contents in natural or engineered plants are low, and the extraction processes are high-cost. Moreover, many medicinal plants grow slowly, and the planting area is limited due to the climate and other environmental conditions (Wang J. et al., 2015). The structure of most monoterpenoids is complex, thus, chemical synthesis is difficult and high-cost (Fu et al., 2018). Therefore, other sustainable supply of monoterpenoids is of great interest.

The development of synthetic biology and metabolic engineering provides opportunities for the microbial biosynthesis of monoterpenoids (Chandran et al., 2011). Industrial-scale production of some terpenoids using engineered microorganisms have been realized, such as artemisinin acid and ginsenosides (Paddon et al., 2013; Yan et al., 2014; Wang P. et al., 2015; Wei et al., 2015; Yang et al., 2020). Recovery of the monoterpenoid biosynthetic pathways and introduction of essential key enzymes into proper microbial host can lead to the production of targeted monoterpenoids (Zebec et al., 2016; Ignea et al., 2019). These provide an alternative way to sustainably supply of monoterpenoids and other Chinese herb-derived natural product (Guan et al., 2020; Wang et al., 2020).

### The Function of Plant-Derived Terpenoids

Plant-derived terpenoids (also known as isoprenoids) are secondary metabolites, and they are widely used in the cosmetic, pharmaceutical, fragrance, and flavor industries (Goto et al., 2010; Caputi and Aprea, 2011). The basic skeleton of plant-derived terpenoids are the five-carbon unit of isoprene. According to the number of isoprene unit, terpenoids are classified into monoterpenoids, sesquiterpenoids, diterpenoids, triterpenoids, etc. (Ashour et al., 2010). Plant-derived terpenoids have diverse biological properties, for example, artemisinin (sesquiterpenoid) has anti-malarial effects (Talman et al., 2019); Taxol (diterpenoid) is used to treat ovarian cancer and breast cancer (Weaver, 2014); Glycyrrhetinic acid (triterpenoid) has antiviral and antimicrobial function (Kowalska and Kalinowska-Lis, 2019); Lycopene (tetraterpenoid) has antioxidant effects (Costa-Rodrigues et al., 2018) and helps reduce the risk of osteoporosis (Oliveira et al., 2019).

Many medicinal plants contain volatile monoterpenoids (Zhu et al., 2020). Among them, menthone and pulegone have anti-inflammatory and antiviral pharmacological effects (Božović and Ragno, 2017); (+)-Menthol and other monoterpenoids have antibacterial effects on *Staphylococcus aureus* and *Escherichia coli* (Trombetta et al., 2005). Monoterpenoids have been used as ingredients of soap, perfume and food, and some monocyclic monoterpenes can be used as insecticides (Rajput et al., 2018).

### Terpenoid Production Strategies

Currently, three possible terpenoid production strategies have been applied, including plant extraction, chemical synthesis, and microbial biosynthesis (Li et al., 2020). Extraction from biomass of herb plants is the traditional terpenoid production method. The monoterpenoids of menthone and pulegone are the effective medicinal ingredients of *Nepeta cataria* (Wang et al., 2017). The volatile components of *N. cataria* are mainly extracted by the physical methods of distillation and extraction, including reflux method, and temperature-programmed microwave extraction method (Li et al., 2012). However, the volatile oil contents in *Nepeta* are low, and extraction of monoterpenoids from *Nepeta* biomass is difficult (Wen et al., 2019). The chemical structures of terpenoids are relatively complex. Chemical synthesis of terpenoids needs many steps, and the purity and yield of the products are not high enough, making chemical synthesis of terpenoids is difficult and high-cost. With the development of synthetic biology and metabolic engineering, many terpenoids have been successfully synthesized by engineered microorganisms (Sun et al., 2017; Guan et al., 2020). The most successful example is the synthesis of artemisinic acid using metabolically engineered yeasts (Paddon et al., 2013; Kai et al., 2018). In China, the most successful large-scale production of terpenoids is the synthesis of rare ginsenosides with engineered *S. cerevisiae* (Yan et al., 2014; Wang P. et al., 2015; Wei et al., 2015). Due to the complex structures of monoterpenoids, microbial production of monoterpenoids is of great interest.

### Recovery of Monoterpenoid Biosynthetic Pathway

**FIGURE 1:**
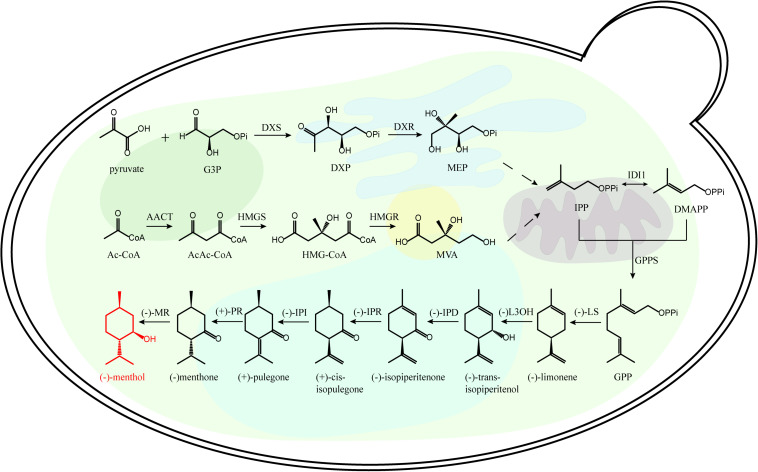
Representative monoterpenoid (menthone) biosynthetic pathway. Ac-CoA, acetyl-CoA; DMAPP dimethylallyl diphosphate; DXP, 1-deoxy-D-xyulose-5-phosphate; DXR, 1-deoxy-D-xylulose 5-phosphate reductoisomerase; DXS, 1-deoxy-D-xylulose 5-phosphate synthase; G3P, glyceraldehyde 3-phosphate; GPP, geranyl diphosphate; GPPS, GPP synthase; HMG-CoA, 3-hydroxy-3-methylglutaryl-CoA; HMGR, 3-hydroxy-3-methylglutaryl-CoA reductase; IDI1, isopentenyl diphosphate isomerase; IPD, *trans-*isopiperitenol dehydrogenase; IPI, *cis-*isopulegone isomerase; IPR, isopiperitenone reductase; L3OH, limonene-3-hydroxylase; LS, limonene synthase; MEP, 2C-methyl-D-erythritol 4-phosphate; MR, menthone reductase; MVA, mevalonate; PR, pulegone reductase.

The recovery of monoterpenoid biosynthetic pathway is essential for microbial monoterpenoid biosynthesis. The carbon skeleton of monoterpenoid is formed by the condensation of isopentenyl pyrophosphate (IPP) and dimethylallyl pyrophosphate (DMAPP) (Wang, 2013). There are two distinct biochemical pathways for the synthesis of IPP and DMAPP, the 2C-methyl-D-Erythritol-4-phosphate (MEP) pathway and the mevalonic acid (MVA) pathway (Figure 1 and Supplementary Figure 1). In the plastids of plant cells, protists and most microorganisms, MEP pathway is used to synthesize IPP and DMAPP; While in the cytoplasm of higher eukaryotes, the MVA pathway is used to synthesize IPP and DMAPP (Laule et al., 2003; Vranová et al., 2013).

There are several metabolic rate-limiting enzymes in MVA and MEP pathways. In MVA pathway, 3-hydroxy-3-methyl glutaryl coenzyme A reductase (HMGR) is a rate-limiting enzyme, which catalyzes HMG-CoA to form MVA (Asadollahi et al., 2010); In MEP pathway, 1-deoxy-D-xyulose-5-phosphate synthase (DXS) is the rate-limiting enzyme, which catalyzes the formation of branched MEP using 1-deoxy-D-xyulose-5-phosphate (DXP) (Bergman et al., 2019). IPP and DMAPP are generated by the MEP pathway or MVA pathway, then they are catalyzed by GPPS (geranyl diphosphate synthase) to form geranyl diphosphate (GPP) with C10 backbone. Diverse terpene synthases (TPSs) catalyze the production of different monoterpenoids using the acyclic monoterpene precursor of GPP (Liao et al., 2016).

Some monoterpenoids exhibit significant toxicity to bacteria by interfering with bacterial cell membranes. Besides, the accumulation of terpenoids precursors (such as IPP) has a negative impact on bacterial growth (Paramasivan and Muttur, 2017). Production of monoterpenoids often needs to express cytochrome P450 genes, which is not easy to achieve in bacteria. In view of these, *S*. *cerevisiae* is becoming an attractive host for monoterpenoid bio-production, due to its robustness, well-studied genetic background, applicability to industrial bioprocesses, and the possibility for terpene scaffold decoration by the functional expression of cytochrome P450 enzymes (Zhang et al., 2008). In particular, *S*. *cerevisiae* is classified as generally regarded as safe (GRAS) by the U.S. Food and Drug Administration (FDA) (Nevoigt, 2008). However, compared with plant cells, *S. cerevisiae* lacks monoterpene synthetase, so it has no ability to synthesize monoterpenoids directly.

In order to realize the heterologous expression of target monoterpenoids in *S. cerevisiae*, exogenous plant monoterpene synthetases need to be introduced (Figure 1). Dudareva et al. (1996) identified one plant monoterpene synthetase gene responsible for the synthesis of terpene alcohol synthase. Chen et al. (2010) isolated and identified two monoterpene synthetases of AaLS1p and ApLS1p, from *Actinidia argute* and *Actinidia polygama*, respectively, which can synthesize (*S*)-linalool. Oswald et al. (2007) co-expressed linalool synthase from *Clarkia breweri* and geraniol synthetase from *Ocimum basilicum* in *S. cerevisiae*, and these enzymes improved the biosynthetic ability of monoterpene linalool and geraniol in *S. cerevisiae*. Herrero et al. (2008) successfully introduced linalool biosynthetic pathway to *S. cerevisiae*, and linalool was effectively produced in *S*. *cerevisiae*. These studies showed that *S. cerevisiae* has great potential to act as a cell factory for monoterpenoids synthesis.

Recently, transcriptomic analyses of the mint adenoids successfully identified the key enzymes of menthol biosynthetic pathway, further cloning and expression verified the biological functions of these key enzymes in the GPP to (−)-menthol pathway (Figure 1 and Supplementary Figure 1; Vining et al., 2017). Different chiral terpenoids often have different biological activities (Hajagos-Tóth et al., 2015), and the key enzymes involved in different terpenoid biosynthetic pathway need to be identified. Therefore, global omics analyses of different monoterpenoid producing plants will give insights into key enzymes for diverse monoterpenoid biosynthesis using engineered microbial cell factories (Wei et al., 2019).

### Applications of Protein Engineering, Metabolic Engineering and Structure Biology for Monoterpenoid Production in *S. cerevisiae*

Although *S. cerevisiae* has been proven to be able to synthesize monoterpenoids efficiently, the metabolic flux of MVA pathway in *S. cerevisiae* is still low (Ignea et al., 2019). The metabolic regulation mechanism of the MVA pathway is complex, which greatly affects the final production of monoterpenoids. In order to increase the metabolic flux of the MVA pathway, several important metabolic rate-limiting/key enzymes have been identified and optimized.

3-hydroxy-3-methyl glutaryl coenzyme A reductase is a rate-limiting enzyme in monoterpenoid synthesis, which catalyzes HMG-CoA to generate MVA irreversibly (Figure 1). To relieve the intermediate product inhibition and accumulate of end products, the N-terminal membrane of HMGR was anchored and the C-terminal of HMGR separately (tHMGR) was expressed in *S. cerevisiae*, which increased the final production of target monoterpenoid (Rico et al., 2010). Overexpression of *tHMGR* and other genes in *S. cerevisiae* lead to 22.49 mg/L limonene (Wu et al., 2018). HMGR2 is the predominant isoenzyme that catalyzes HMG-CoA reduction under hypoxia condition (Mantzouridou and Tsimidou, 2010), an engineered stabilized mutant HMGR2 (K6R) successfully improved the production of terpenes (Supplementary Figure 2; Ignea et al., 2012, 2014). These protein engineering studies suggested that HMGR had a significant effect on target monoterpenoids production.

Isoprene pyrophosphate isomerase (IDI1) controls the first step of the monoterpenoid biosynthesis in the conversion process from IPP to DMAPP, which can adjust the proportion of IPP/DMAPP (Figure 1; Cao et al., 2016). Regulating the expression of *IDI1* successfully increased the yield of monoterpenoid. Further overexpression of *IDI1* gene in engineered *S. cerevisiae* increased sabinene production by 300% (Ignea et al., 2014). These examples indicate that overexpression of *IDI1* in *S. cerevisiae* could increase the production of desirable monoterpenoid.

**FIGURE 2:**
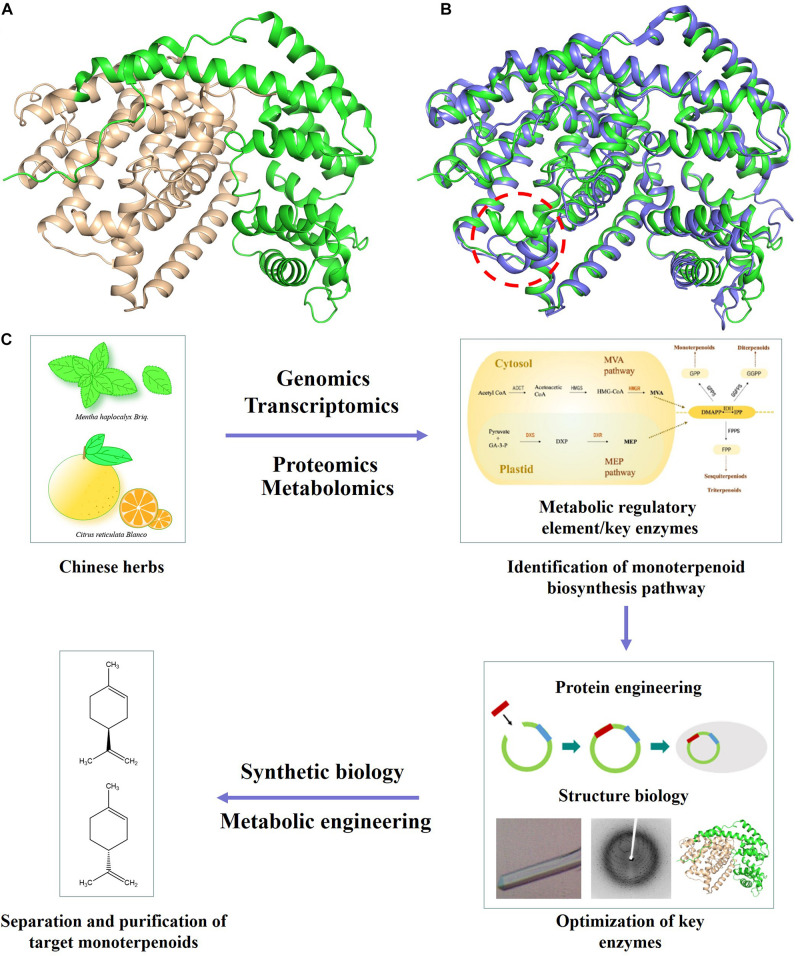
Application of protein engineering and structural biology based approaches in yeast monoterpenoid production. **(A)** Structure of (–)-LS (PDB ID 2ONG). N-terminal and C-terminal domain are colored in green and wheat, respectively. **(B)** Comparison of the structure of (–)-LS (colored in green) and (+)-LS (colored in blue). **(C)** Increasing the production of target monoterpenoid through optimizing important metabolic nodes/key enzymes by enzyme engineering or structure biology.

The committed step in monoterpenoid biosynthesis involves the conversion of the acyclic isoprenoid diphosphate precursor to cyclic hydrocarbon product catalyzed by limonene synthase (LS) (Figure 1 and Supplementary Figure 1). Limonene is well known for its olfactory characteristics and antibacterial activity, which is often added to soaps or detergents (Jongedijk et al., 2016). Limonene has an unsaturated ring structure, which can be oxidized to menthol, perillyl alcohol and other natural products (Alonso-Gutierrez et al., 2013). Expression of a (−)- limonene synthase from *Perilla frutescens* and a (+)-limonene synthase from *Citrus sinensis* resulted in the production of 0.028 mg/l (+)-limonene and 0.060 mg/l (−)-limonene in the engineered yeast cell factory (Jongedijk et al., 2014). The crystal structure of (−)-limonene synthase [(−)-LS] from *Mentha spicata* was determined at 2.7 Å resolution. The structural and biochemical results show that (−)-LS shares many hallmark features and catalytic characteristic with plant monoterpene synthases, including an all-α-helical domain secondary structure, a two-domain architecture with a catalytic C-terminal domain, an N-terminal domain of unknown function, and conserved divalent metal ion binding residues in the active site (Figure 2A; Hyatt et al., 2007).

(+)-Limonene is an abundant monoterpene in the essential oils of most citrus fruit, which is commonly used as an industrial and household solvent. In particular, the citrus smell enables its use in the fragrance and flavoring industries. The (+)-LS from *Citrus sinensis* was purified, and its crystal structure was further determined. The structural comparison of (+)-LS and (−)-LS showed that the conformation of short α1 helix played critical roles in determining (+)-LS or (−)-LS enantiomer. The structural and catalytic mechanism studies of (+)-LS and (−)-LS provided theoretical basis and new options for designing and achieving (+) or (−)-limonene rationally (Figure 2B; Morehouse et al., 2017; Ren et al., 2020). Based on the LS crystal structures and the proposed catalytic mechanism, a triple mutant (S454G, C457V, M458I) of *Mentha spicata* (−)-LS was successfully generated, which showed a new catalytic activity of producing more complex bicyclic monoterpenes (Supplementary Figure 2; Leferink et al., 2018).

Farnesyl pyrophosphate synthase (FPPS) condenses one molecule of DMAPP and one molecule of IPP to form GPP, then condenses GPP with one molecule of IPP to form FPP in *S. cerevisiae* (Figure 1). It is reported that FPPS of *S. cerevisiae* is encoded by the gene *ERG20*, which is responsible for the biosynthesis of GPP and FPP. GPP is usually closely bound to the catalytic pocket of FPPS and results in the low content of free GPP (Dai et al., 2013). Therefore, the precursor GPP for monoterpenoid synthesis was insufficient, which greatly restricted the biosynthesis of monoterpenoids. In addition, *S. cerevisiae* also lacks specific and efficient GPP synthase (Herrero et al., 2008). Sequence alignment of FPPS from different organisms showed that there were two ASP rich sequences in the conserved region of this enzyme. The first ASP rich sequence is DDXXD or DDXXXXD, which could bind DMAPP, GPP, and FPP in catalytic reaction. The second ASP rich sequence is DDXXD, which is mainly responsible for binding IPP. Previous studies showed that site directed mutagenesis in the conservative region of FPPS can cause GPP released from the binding site of FPPS, which increased the intracellular free GPP content (Supplementary Figure 2). By mutating the catalytic residue K197 of yeast farnesyl diphosphate synthase erg20p and expressing heterologous geraniol synthetase gene in engineered *S. cerevisiae*, the yield of geraniol increased significantly, suggesting that the regulation of *ERG20* gene can increase the GPP amount and balance GPP and FPP ratio for geraniol production (Fischer et al., 2011). Sabinene is seen as a potential component for the next generation of aircraft fuels (Rude and Schirmer, 2009). Ignea et al. (2014) identified that erg20p is an rate-limiting enzyme for monoterpene production, and integrating erg20p with a geranyl diphosphate synthase increased sabinene titer (17.5 mg/L). Compared with the canonical GPP-based monoterpenoid biosynthetic pathway, a synthetic orthogonal monoterpenoid pathway using neryl diphosphate as substrate was designed, and further dynamic regulation of some engineered enzymes in the synthetic monoterpenoid improved monoterpenoid production by seven-fold (Ignea et al., 2019).

The protein engineering and structural studies of key enzymes involved in monoterpenoid biosynthetic pathway provided new options for efficient monoterpenoid biosynthesis in *S. cerevisiae* (Figure 2C and Supplementary Figure 2).

### Conclusion and Future Perspective

Plant-derived monoterpenoids are widely used in daily life, and the demand is increasing. Microbial biosynthesis of monoterpenoid provides a new feasible strategy for the monoterpenoid production. *S. cerevisiae* might be an ideal microbial cell factory for monoterpenoid biosynthesis. With the help of omics technologies, systems biology, structural biology and protein engineering, it will further lead to the identification of highly efficient monoterpenoid biosynthetic enzymes, which can help to enable high yield of monoterpenoid in *S. cerevisiae*.

## Author Contributions

QG, LW, and MZ contributed to figures and manuscript draft preparations. YW and WL wrote and revised the manuscript. WL conceived the project. All authors approved it for publication.

## Conflict of Interest

The authors declare that the research was conducted in the absence of any commercial or financial relationships that could be construed as a potential conflict of interest.
