# Supplementary material for: Recent Advances on Feasible Strategies for Monoterpenoid Production in Saccharomyces cerevisiae
Source: Front Bioeng Biotechnol. 2020 Dec 1;8:609800. doi: 10.3389/fbioe.2020.609800 (PMC7736617; doi:10.3389/fbioe.2020.609800)
Supplement: Supplementary file 1 [file Data_Sheet_1.PDF]

Recent advances on feasible strategies for monoterpenoid production in *Saccharomyces cerevisiae*

Qiyu Gao[1,2,3#], Luan Wang[4#], Maosen Zhang[5#], Yongjun Wei[4*], Wei Lin[1,2,3*]

[1]Department of Microbiology and Immunology, School of Medicine & Holistic Integrative Medicine, Nanjing University of Chinese Medicine, Nanjing, China.

[2]State Key Laboratory of Natural Medicines, China Pharmaceutical University, Nanjing, China

[3]Jiangsu Collaborative Innovation Center of Chinese Medicinal Resources Industrialization, Nanjing 210023, China.

[4] Key Laboratory of Advanced Drug Preparation Technologies, Ministry of Education & School of Pharmaceutical Sciences, Zhengzhou University, Zhengzhou, 450001, China;

[5]Affiliated Hospital of Nanjing University of Chinese Medicine, Jiangsu Province Hospital of Chinese Medicine, Nanjing, China.

[#]These three authors contributed equally to this work.

#, Equal contribution.

*** Correspondence:**
Corresponding Authors
weilin@njucm.edu.cn and yongjunwei@zzu.edu.cn

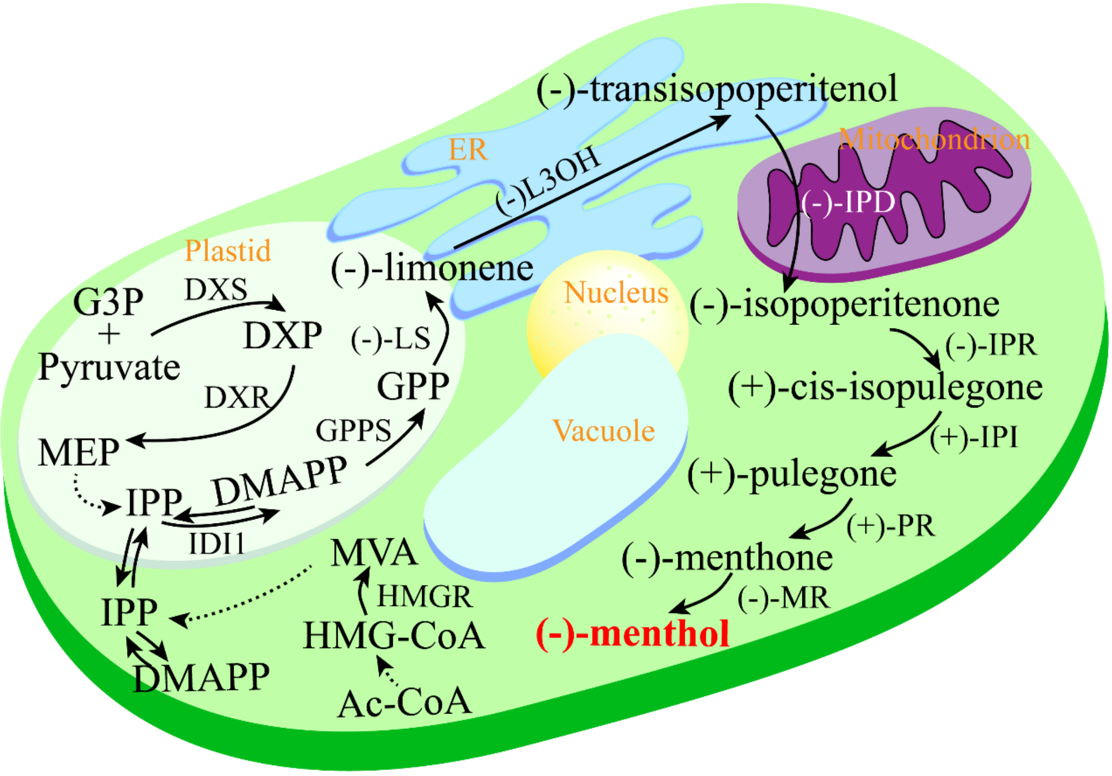

Supplementary Figure S1. Representative monoterpenoid (menthone) biosynthetic pathway in plants. Ac-CoA, acetyl-CoA; DMAPP dimethylallyl diphosphate; DXP, 1-deoxy-D-xyulose-5-phosphate; DXR, 1-deoxy-D-xylulose 5-phosphate reductoisomerase; DXS, 1-deoxy-D-xylulose 5-phosphate synthase; G3P, glyceraldehyde 3-phosphate; GPP, geranyl diphosphate; GPPS, GPP synthase; HMG-CoA, 3-hydroxy-3-methylglutaryl-CoA; HMGR, 3-hydroxy-3-methylglutaryl-CoA reductase; IDI1, isopentenyl diphosphate isomerase; IPD, trans-isopiperitenol dehydrogenase; IPI, cis-isopulegone isomerase; IPR, isopiperitenoe reductase; L3OH, limonene-3-hydroxylase; LS, limonene synthase; MEP, 2C-methyl-D-erythritol 4-phosphate; MR, menthone reductase; MVA, mevalonate; PR, pulegone reductase.

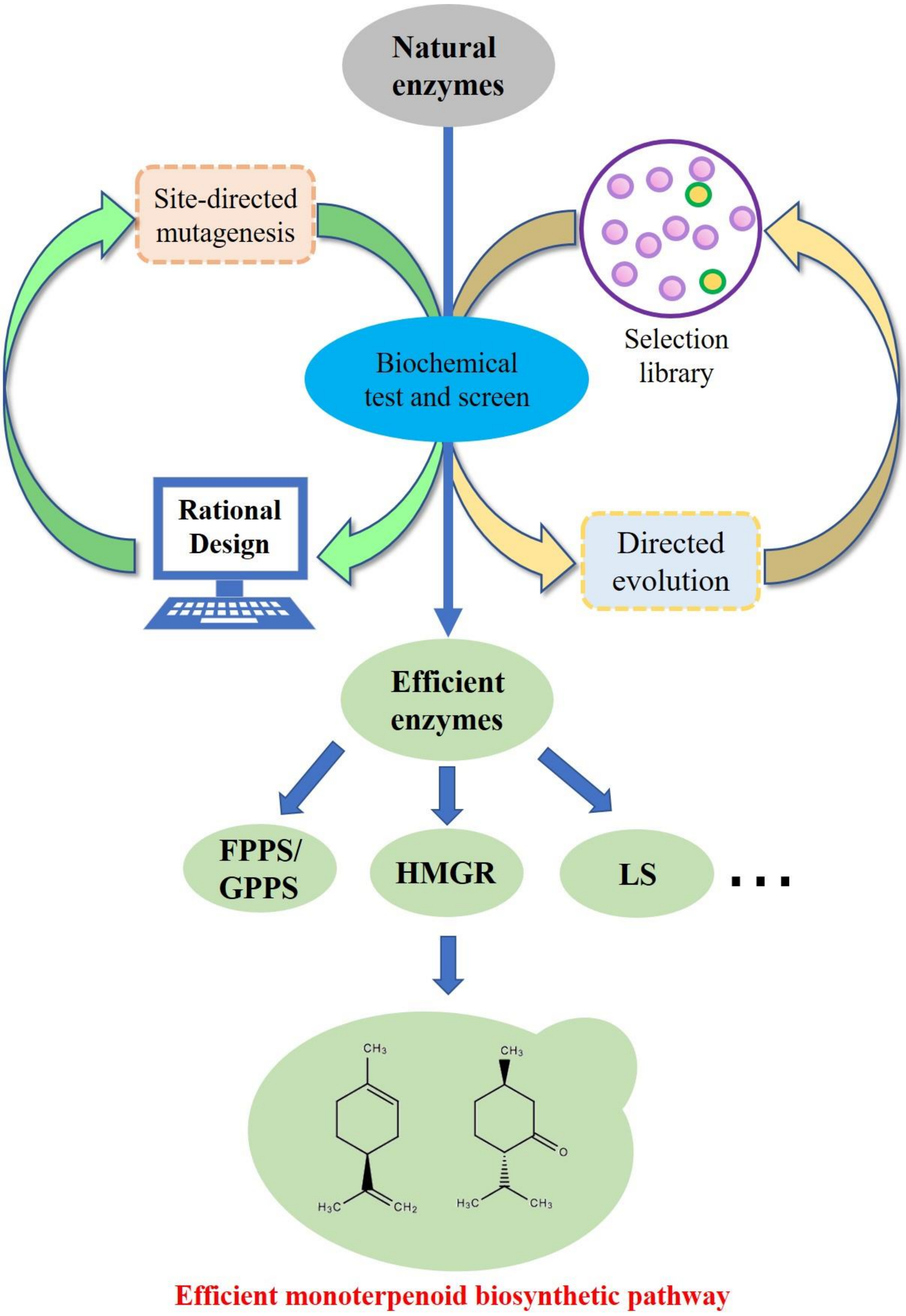

Supplementary Figure S2. Protein engineering for efficient enzyme screening. The two main strategies of rational design and directed evolution were used for protein engineering until efficient enzymes were obtained. The efficient enzymes may comprise efficient biosynthetic pathway for terpenoid production.
